# Current News

**Published:** 1893-02

**Authors:** 


### Current News.

### WORLD'S COLUMBIAN DENTAL CONGRESS.

### STANDING COMMITTEES.

The following additions have been made to the Committees of the Congress, and are to be added to the list as published in the International Dental Journal for June, 1892, page 475:

*Committee on Conference.*—C. N. Peirce, Philadelphia, Pa.; A. Warner, Jr., San Francisco, Cal.; George H. Cushing, Chicago, Ill.; J. N. Crouse, Chicago, Ill.; W. Herbst, Bremen, Germany; Wm. Jarvie, Brooklyn, N. Y.; R. E. Watkins, Eutaw, Ala.; S. B. Brown, Fort Wayne, Ind.; S. A. Main, New York City, N. Y.; H. A. Smith, Cincinnati, Ohio; C. R. Butler, Cleveland, Ohio; Chas. J. Essig, Philadelphia, Pa; James Truman, Philadelphia, Pa.; Garrett Newkirk, Chicago, Ill.; A. R. Eaton, Elizabeth, N. J.; W. J. Younger, San Francisco, Cal.; H. M. Hunter, San Antonio, Texas; W. R. Patton, Cologne, Germany; F. H. Balkwill, Plymouth, England; R. T. Stack, Dublin, Ireland; W. B. Pearsall, Dublin, Ireland; Henry Sewill, London, England; B. A. Muckenfuss, Charleston, S. C.; W. E. Magill, Erie, Pa.; C. C. Chittenden, Madison, Wis.; Frank Abbott, New York City, N. Y.; C. E. Francis, New York City, N. Y.; J. L. Williams, Boston, Mass.; E. A. Bogue, New York City, N. Y.; P. G. C. Hunt, Indianapolis, Ind.; J. E. Cravens, Indianapolis, Ind.; E. H. Angle, Minneapolis, Minn.; Ludwig Holländer, Halle, Germany; W. Campbell, Dundee, Scotland; S. B. Cook, Chattanooga, Tenn.; W. T. Arrington, Memphis, Tenn.; B. G. Maercklein, Milwaukee, Wis.; A. W. Nason, Omaha, Neb.; S. J. Barber, Portland, Ore.; C. S. Case, Jackson, Mich.; L. C. Ingersoll, Keokuk, Iowa; Wm. Taft, Cincinnati, Ohio; J. Hayhurst, Lambertville, N. J.; A. O. Rawls, Lexington, Ky.; J. N. Farrar, New York City, N. Y.; E. T. Darby, Philadelphia, Pa.; G. W. Rembert, Natchez, Miss.; Louis Augspath, Little Rock, Ark.; W. G. A. Bonwill, Philadelphia, Pa.; T. B. Welch, Vineland, N. J.; Geo. Watt, Xenia, Ohio; O. E. Hill, Brooklyn, N. Y.; M. Delapierre, Brussels, Belgium; C. Van der Hoeven, Haag, Holland; R. Skogsburg, Stockholm, Sweden; Julius Scheff, Vienna, Austria; Wm. Alfred Hunt, Somersetshire, England; C. F. Rich, Saratoga, N. Y.; Wm. Edward Harding, Shrewsbury, England; James Leslie Fraser, Inverness, Scotland; Julius Parreidt, Leipzig, Germany; Carl Kaas, Christiania, Norway; V. E. Turner, Raleigh, N. C.; A. A. Dillehay, Meridian, Miss.; J. J. Andrew, Belfast, Ireland.

*No.* 2. *Programme Committee.*—Chairman, C. N. Johnson, Chicago, Ill.; J. A. Dunn, Chicago, Ill.; Geo. Eubank, Birmingham, Ala.; J. W. Wassall, Chicago, Ill.; Geo. Hardy, Baltimore, Md.; Louis Ottofy, Chicago, Ill.; L. P. Bethel, Kent, Ohio; E. T. Rippier, Brooklyn, N. Y.

*No.* 3. *Committee on Exhibits.*—M. L. Rhein, New York City, N. Y.; A.

W. McCandless, Chicago, Ill.; R. C. Young, Anniston, Ala.; James Chace, Ocala, Fla.; W. A. Campbell, Brooklyn, N. Y.

*No.* 4. *Committee on Transportation.*—John C. Storey, Dallas, Texas; H. A. Fynn, Denver, Col.

*No.* 5. *Committee on Reception.*—Chairman, J. A. Swasey, Chicago, Ill.; E. D. Swain, Chicago, Ill.; M. V. Toledo, New York City, N. Y.; A. E. Baldwin, Chicago, Ill.; A. F. Emminger, Columbus, Ohio; T. L. Gilmer, Chicago, Ill.; H. J. Burkhart, Batavia, N. Y.; F. N. Browne, Abilene, Texas; J. W. Taylor, New York City, N. Y.; Geo. L. Field, Detroit, Mich.; F. E. Howard, Buffalo, N. Y.; A. H. Fuller, St. Louis, Mo.; H. W. Shriver, Omaha; Neb.; R. K. Luckie, Holly Springs, Miss.; C. S. Searles, Dubuque, Iowa; L. D. Shepard, Boston, Mass.; J. P. Geran, Brooklyn, N. Y.; R. M. Streeter, New York City, N. Y.; Thos. E. Weeks, Minneapolis, Minn.; Chas. H. Darby, St. Joseph, Mo.

*No.* 6. *Committee on Registration.*—W. W. Hill, Washington, Ga.; Chas. L. Dubar, New York City, N. Y.; S. W. Foster, Decatur, Ala.; H. N. Young, Wilkes-Barre, Pa.; G. Carleton Brown, Elizabeth, N. J.

*No.* 7. *Committee on Printing Transactions.*—A. W. Harlan, Chicago, Ill.; Edward C. Kirk, Philadelphia, Pa.; Louis Ottofy, Chicago, Ill.

*No.* 8. *Committee on Conference with State and Local Societies.*—Chairman, S. A. Mulkey, Moscow, Idaho; Chas. A. Meeker, Newark, N. J.; J. R. Callahan, Cincinnati, Ohio; J. R. Cardwell, Portland, Ore.; George H. Ames, Providence, R. I.; L. D. Shepard, Boston, Mass.; Geo. A. Maxfield, Holyoke, Mass.; James H. Daly, Boston, Mass.; F. C. Walker, Brooklyn, N. Y.; Wm. Smedley, Denver, Col.; Wm. Garnett, Pueblo, Col.; W. E. Walker, Bay St. Louis, Miss.; C. H. Harroun, Toledo, Ohio; S. B. Cook, Chattanooga, Tenn.; R. J. Gardinier, Laramie, Wyo.; A. F. Thode, Rawlins, Wyo.

*No.* 9. *Committee on the History of Dental Legislation in this and other Countries.*—C. Stoddard Smith, Chicago, Ill.; S. R. Salazar, Lima, Peru; W. F. Rehfuss, Philadelphia, Pa.; Theo. Frisch, Zurich, Switzerland; E. Palmer, La Crosse, Wis.; G. W. Copp, Durango, Col.; Walter S. Leaming, Cape May, N. J.; R. A. Holliday, Atlanta, Ga.

*No.* 11. *Committee on Invitation.*—Thos. Fillebrown, Boston, Mass.; Thos. T. Moore, Columbia, S. C.; Geo. B. Steel, Richmond, Va.

*No.* 12. *Committee on Membership.*—E. L. Townsend, Los Angeles, Cal.; C. E. Hussey, Biddeford, Me.; J. J. Pitts, Brooklyn, N. Y.; J. Frank Mariner, Chicago, Ill.

*No.* 13. *Committee on Education and Literary Exhibits.*—D. M. Sabater, New York City, N. Y.; H. G. Mirick, Brooklyn, N. Y.

*No.* 14. *Committee on Clinics in Operative Dentistry and Oral Surgery.*—J. S. Marshall, Vice-Chairman, Chicago, Ill.; T. L. Gilmer, Chicago, Ill.; S. C. G. Watkins, Mont Clair, N. J.; M. C. Gottschaldt, New York City, N. Y.; R. Heide, Paris, France; J. G. Reid, Chicago, Ill.; B. D. Wikoff, Chicago, Ill.; F. T. Breene, Iowa City, Iowa; N. S. Hoff, Ann Arbor, Mich.; J. H. Gaskill, Philadelphia, Pa.; J. M. Norman, Denver, Col.; James Goodwillie, New York City, N. Y.; G. S. Salomon, Chicago, Ill.; G. V. I. Brown, Duluth, Minn.; P. T. Smith, Denver, Col.

No. 15. *Committee on Prosthetic Dentistry.*—Harry Rose, London, England; F. H. Balkwill, Plymouth, England; J. R. Knapp, New Orleans, La.;

C. M. Richmond, New York City, N. Y.; W. B. Ames, Chicago, Ill.; G. Molyneaux, Cincinnati, Ohio; C. V. Rosser, Atlanta, Ga.; John S. Thompson, Atlanta, Ga.; F. M. Shriver, Glenwood, Iowa; M. A. Bartleson, Denver, Col.; C. H. Darby, St. Louis, Mo.; W. H. Coffin, London, England; W. Mitchell, London, England.

*No.* 17. *Committee on Essays.*—C. N. Peirce, Philadelphia, Pa.

*No.* 18. *Committee on History of Dentistry in the United States.*—J. Hayhurst, Lambertville, N. J.; F. H. Sutherland, Pueblo, Col.

*No.* 19. *Committee on Nomenclature.*—Chairman, G. V. Black, Jacksonville, Ill.; E. C. Kirk, Philadelphia, Pa.; W. O. Kulp, Davenport, Iowa; J. Edwin Line, Rochester, N. Y.; J. B. Hodgkin, Washington, D. C.; J. S. Cassidy, Covington, Ky.; C. T. Stockwell, Springfield, Mass.; A. Witzel, Essen, Germany; E. Richter, Berlin, Germany; J. von Metnitz, Vienna, Austria; O. Rosenthal, Liege, Belgium; P. Sidney Spokes, London, England; E. Damain, Paris, France; H. J. Billeter, Zurich, Switzerland; O. Amoedo, Paris, France; A. C. Hugenschmidt, Paris, France.

*No.* 20. *Committee to promote the Appointment of Dental Surgeons in the Armies and Navies of the World.*—Frank Woodbury, Halifax, Nova Scotia; Thos. Gaddes, London, England; A. H. Brockway, Brooklyn, N. Y.; J. Bond Littig, New York City, N. Y.

*No.* 21. *Committee on Care of the Teeth of the Poor.*—Chairman, T. H. Parramore, Hampton, Va.; M. V. Johnson, Holden, Mo.; J. Allen Osmun, Newark, N. J.; C. S. Butler, Buffalo, N. Y.; Corydon Palmer, Warren, Ohio; Geo. E. Adams, South Orange, N. J.; H. M. Smith, Colorado Springs, Col.

*No.* 22. *Committee on Microscopy and Bacteriology.*—G. V. Black, Jacksonville, Ill.; Richard Chauvin, Paris, France.

*No.* 23. *Committee on Prize Essays.*—E. P. Keech, Baltimore, Md.; Otto Arnold, Columbus, Ohio; C. B. Rohland, Alton, Ill.

*No.* 24. *Editorial Committee.*—A. W. Harlan, Chicago, Ill.

*Sub-Committee.*—A. W. Harlan, Chicago, Ill.; A. O. Hunt, Iowa City, Iowa; John S. Marshall, Chicago, Ill.

REPORT OF COMMITTEE ON FOREIGN HONORARY OFFICERS.

*Great Britain.*—Honorary President, J. H. Mummery, London, England. Honorary Vice-Presidents, William Herbert Woodruff, London, England; W. B. Macleod, Edinburgh, Scotland; W. B. Pearsall, Dublin, Ireland; F. H. Balkwill, Plymouth, England. Honorary Secretary, Geo. Cunningham, Cambridge, England.

*Canada.*—Honorary President, W. Geo. Beers, Montreal. Honorary Vice-Presidents, A. C. Cogswell, Halifax, Nova Scotia; J. B. Willmot, Toronto. Honorary Secretary, R. H. Robertson, Portage La Prairie, Manitoba.

*Holland.*—Honorary President, John E. Grevers, Amsterdam. Honorary Vice-Presidents, A. C. J. Koenaart, Rotterdam; Theo. Dentz, Utrecht.

*Germany.*—Honorary President, Dr. Robert Carl Franz, Berlin. Honorary Vice-Presidents, Dr. Ludwig Holländer, Halle; F. W. Herbst, Bremen; Julius Parreidt, Leipzig; Wilhelm Sachs, Breslau; F. Hesse, Leipzig; Adolph Witzel, Essen. Honorary Secretary, Ludwig Warnekros, Berlin.

*Belgium.*—Honorary President, H. Bon, Brussels. Honorary Vice-Presidents, J. Verschuren, Antwerp; J. Binge, Liege; L. C. Depaepe, Louvain; Hect. Minne, Antwerp; J. F. Pourveur, Antwerp. Honorary Secretary, Dr. von Blairen, Brussels.

*France.*—Honorary President, E. Lecaudey, Paris. Honorary Vice-Presidents, V. Anjubault, Paris; Henry Crignier, Paris; M. F. Touchard, Paris; Charles Godon, Paris; V. Galippe, Paris. Honorary Secretary, F. Ducournau, Paris.

*Switzerland.*—Honorary President, C. O. Schulthess, Basel. Honorary Vice-Presidents, P. A. Kölliker, Zurich; Louis Roussy, Geneva. Honorary Secretary, Paul Witzig, Basel.

*Austria-Hungary.*—Honorary President, H. Schmid, Prague. Honorary Vice-Presidents, Josef Izlai, Budapest; Julius Scheff, Jr., Vienna; Carl Fischer-Colbric, Vienna; Anton Bleichsteiner, Gratz; W. Vajna, Siebenburgen. Honorary Secretary, Anton Papsch, Innspruck.

*Roumania.*—Honorary President, L. Goldberge, Galatz. Honorary Vice-Presidents, Dr. Lempart, Bucharest; P. Macarowic, Jassy; M. End, Bucharest.

*Spain.*—Honorary President, Francisco Carbonell, Barcelona. Honorary Vice-Presidents, Pirso Perez, Madrid; M. Trallero, Barcelona; A. Trivino, Madrid; R. H. Portuondo, Madrid; Y. de Otaola, Bilboa. Honorary Secretary, Florestan Aguilar, Cadiz.

*Portugal.*—Honorary President, Y. P. G. Pavia, Lisbon. Honorary Vice-Presidents, Carl Koth, Lisbon; J. R. Da Silva, Oporto.

*Italy.*—Honorary President, Cesare Campani, Florence. Honorary Vice-Presidents, Pietro Ribolla, Rome; Sig. Cav. Dott Francisco Garelli, Turin; Carlo Platschick, Milan; Antonio Damiano Mela, Genoa; Luigi Ribolla Nicodemi, Palermo. Honorary Secretary, Giuseppe Cali, Rome.

*Greece.*—Honorary President, D. Caracatsanis, Athens. Honorary Vice-President, J. Neumann, Athens. Honorary Secretary, to be selected by the President.

*Bulgaria.*—Honorary President, I. A. Muszler, Sofia.

*Servia.*—Honorary President, Dr. Steic, Belgrad.

*Denmark.*—Honorary President, V. Haderup, Copenhagen. Honorary Vice-Presidents, C. Thorlakson, Copenhagen; J. A. Kjartinge, Aarhuus; L. Ed. Fulbius, Copenhagen; S. B. C. Kjaer, Odense. Honorary Secretary, J. L. Secher, Copenhagen.

*Norway.*—Honorary President, A. J. Hovland, Christiania. Honorary Vice-Presidents, M. Andersen, Bergen; O. A. Olsen, Christiansand; H. O. Heide, Christiania; M. H. Beutzen, Bergen. Honorary Secretary, Carl Kaas, Christiania.

*Russia.*—Honorary President, F. J. Washinskij, St. Petersburg. Honorary Vice-Presidents, F. Witas Rhode, Dorpat; Dr. Tyschinski, Odessa; J. J. Chrustchow, St. Petersburg; Antoni Kasprowicz, Warsaw; Paul Adelheim, Moscow; M. Wilhelm Prawedny, St. Petersburg. Honorary Secretary, Ferdinand Klapproth, St. Petersburg.

*Sweden.*—Honorary President, Franz Berggren, Stockholm. Honorary Vice-Presidents, J. A. Bygren, Goteborg; J. Victor Bensow, Goteborg; O. Ulmgren, Stockholm. Honorary Secretary, Elof Forberg, Stockholm.

*Finland.*—Honorary President, Th. Weber, Helsingfors. Honorary Vice-Presidents, Dr. Ayräpää, Helsingfors; Win Olander, Helsingfors.

*South America.*—Honorary Members, J. S. Jenkins, Lima, Peru; Joao Ferreira, Campinas, Brazil; A. Salcedo, Bogota, Colombia; G. Vargas Paredes, Bogota, Colombia; Dr. Caballero, Buenos Ayres; A. W. Isard, Buenos Ayres; W. E. Hill, Montevideo, Uruguay; A. Carballo, Montevideo, Uruguay; J. S. Burrett, Uruguay; Manuel G. Ramos, Guayaquil, Ecuador; Ferman F. Lince, Guayaquil, Ecuador; Raymon F. Coy, Lima, Peru; Dr. Coquet, Buenos Ayres; C. Koth, Rio de Janeiro, Brazil; J. W. Coachman, Rio de Janeiro, Brazil; F. E. Robinson, Iquique, Chili; Juan Estrella, Lima, Peru; Chapot Prevost, Rio de Janeiro, Brazil; Dr. Pereira, Rio de Janeiro, Brazil; Joaquin Camara, Pernambuco, Brazil; Ramon Araya, Santiago, Chili; N. Etcheparaborda, Buenos Ayres; Alex. McG. Denham, Santiago, Chili; Geo. B. Newland, Buenos Ayres; Numa Pompilio, Pernambuco, Brazil; Dr. Ramirez, Valparaiso, Chili.

*Central America.*—Honorary Presidents, Guillermo Hunter, Leon, Nicaragua; Arthur Koehpke, Panama, Isthmus of Panama; J. Luis Estrado, Guatemala City, Guatemala; A. Lacayo, Grenada, Nicaragua.

*Mexico.*—Honorary Presidents, E. Carillo, City of Mexico; E. Bremer, Matamoras; J. W. Bastow, Cohma; M. Warren, City of Mexico; R. J. Rio Rico, City of Mexico; Jose M. Soriano, City of Mexico; Antonio Rodriquez, Mexico; J. K. Foster, Guanajuato; A. H. Wheeler, Oaxaca; C. Cornish, Mexico.

*West Indies.*—Honorary Presidents, Jose Trajillo, Havana, Cuba; Arthur Garesche, Cardenas, Cuba; H. Blackburn Smith, Hamilton, Bermuda; James Gordon, St. Thomas, Danish West Indies; F. T. Crosby, St. John's, Antigua; Dr. Raymond, San Salvador, Central America; Miguel Gutierrez, Havana, Cuba; Juan Mireles, Havana, Cuba; Chas. H. Daly, Trinidad, Isle of Trinidad; P. Aloma Martinez, Trinidad, Cuba; Julio Lyon, San Domingo, Hayti.

*Australia and New Zealand.*—Honorary Presidents, H. H. Norman, Adelaide, South Australia; P. Crank, Adelaide, South Australia; G. W. Trott, Adelaide, South Australia; C. F. Fyffe, East Melbourne, Australia; J. Belisario, Sydney, New South Wales; R. Norman, Auckland, New Zealand; H. P. Rawson, Wellington, New Zealand.

*Africa.*—Honorary Presidents, R. J. Barclay, Cape Town; B. F. Hutchinson, Cape Town; G. W. Baylis, Durban, Natal; J. R. Bill, Grahamstown, South Africa; J. Brown, Kimberly; F. Strickland, Port Elizabeth, South Africa; R. C. Walker, Cairo, Egypt; J. F. Love, Alexandria, Egypt.

*Asia.*—Honorary Presidents, F. E. Daver, Bombay, India; Dr. Bromley, Bombay, India; Dr. Campbell, Bombay, India; Dr. Woods, Calcutta, India; Dr. Ranger, Calcutta, India; A. E. Jones, Lahore, Punjaub, India; L. S. Clark, Bungalore, India; A. O'Mera, Simla, India; Cheong Chun Fin, Singapore, Straits Settlement; R. H. Kimball, Hong-Kong, China; Frank R. Faber, Constantinople, Turkey; D. E. Petersen, Shanghai, China; S. Yenomoto, Tokio, Japan; S. N. Isawa, Tokio, Japan; Hume Purdie, Colombo, Island of Ceylon.

HONORARY OFFICERS OF THE AMERICAN CONTINGENT IN FOREIGN COUNTRIES.

President, Thomas W. Evans, Paris, France. Vice-Presidents, Geo. W. Field, London, England; W. D. Miller, Berlin, Germany; N. S. Jenkins, Dresden, Germany; E. M. Thomas, Vienna, Austria; J. G. Van Marter, Rome, Italy; L. C. Bryan, Basel, Switzerland; A. H. Sylvester, Berlin, Germany; H. C. Edwards, Madrid, Spain; J. M. Whitney, Honolulu, Hawaii; J. Ward Hall, Shanghai, China; I. B. Davenport, Paris, France. Secretary, Frank S. Buckley, Berlin, Germany.

---

### OFFICERS OF THE PHILADELPHIA COUNTY DENTAL SOCIETY.

President, Dr. Frank I. Bassett; Vice-President, Dr. Alonzo Boice; Secretary, Dr. W. A. Phreaner; Treasurer, Dr. Miller.

*Board of Directors.*—Drs. Bassett, Fogg, Abell, Faught, Pike, Guerry, Boice, Miller, and Phreaner.

*Legal Committee.*—Drs. Boice, Faught, and Phreaner.

---

Dr. Chas. H. Taft has removed his office from 273 Oakwood Boulevard to 5401 Jefferson Avenue, Chicago, Ill.

---

### RECENT PATENTS.

A list of recent patents, reported specially for the International Dental Journal:

487,647.—Dental Vulcanizer. George B. Snow, Buffalo, N. Y. Filed August 13, 1892.

487,726.—Dental Regulator. Jonathan A. Ellard, Birmingham, Ala. Filed April 15, 1892.

487,843.—Dental Plugger. Henry R. Kline, Ashtabula, Ohio. Filed April 6, 1892.

487,973.—Dental Separator. Benjamin Simons, Charleston, S. C. Filed June 6, 1892.

488,008.—Method of Forming Dental Crowns. Jeptha G. Hollingsworth, Kansas City, Mo. Filed April 26, 1892.

Trade-Marks.—22,109.—Liquid Anæsthetic for Dentists' Use. George M. Miller, Detroit, Mich. Filed October 3, 1892. Essential feature, the word "Exhedonic."

---

### SAN FRANCISCO DENTAL ASSOCIATION.

At the regular monthly meeting of the San Francisco Dental Association, held in Mystic Hall, Union Square Building, San Francisco, October 10, 1892, the following officers were elected for the ensuing year: L. A. Teague, President; C. E. Post, Vice-President; H. P. Carlton, Recording Secretary; William F. Sharp, Corresponding Secretary; W. A. Knowles, Treasurer; C. E. Post, Librarian.

W. F. Sharp,
*Corresponding Secretary.*

500 Sutter Street, San Francisco.

